# Economic and evolutionary hypotheses for cross-population variation in parochialism

**DOI:** 10.3389/fnhum.2013.00559

**Published:** 2013-09-11

**Authors:** Daniel J. Hruschka, Joseph Henrich

**Affiliations:** ^1^School of Human Evolution and Social Change, Arizona State UniversityTempe, AZ, USA; ^2^Departments of Psychology and Economics, University of British ColumbiaVancouver, BC, Canada

**Keywords:** parochialism, in-group favoritism, cross-cultural, market integration, religion, institutions, parasite stress, closeness

## Abstract

Human populations differ reliably in the degree to which people favor family, friends, and community members over strangers and outsiders. In the last decade, researchers have begun to propose several economic and evolutionary hypotheses for these cross-population differences in parochialism. In this paper, we outline major current theories and review recent attempts to test them. We also discuss the key methodological challenges in assessing these diverse economic and evolutionary theories for cross-population differences in parochialism.

## Introduction

In the last 200 years, the half million Iban living on Borneo's northwest region have undergone a remarkable transformation. When first encountered by colonizers in the 19th century, Iban lived in communal long-houses of 100–200 people and made a living from farming rice and hunting (Freeman, 1970). According to their festivals and mythology, Iban worked toward a community that was harmonious, rich in rice, flush with children, and endowed with an abundance of spiritual energy (Jensen, 1974; Heppell et al., 2005). A key way of fostering such flourishing communities was the taking of human heads—to cure a member of one's group or to rescue a member's soul from limbo or from spiritual slavery in another region (Klokke, 2004). It is important to note here that indiscriminate killing was not acceptable among the Iban. Tribal groupings were defined in part as those people who did not take each other's heads. Killing a fellow group member was considered a major transgression on the order of incest. It could upset the universal order and could lead to sterility in terms of offspring and rice production as well as the future taking of heads (Freeman, 1970; Jensen, 1974; Sutlive, 1992).

Fast forward to today. After the forceful imposition of colonial and state laws banning head-hunting, the practice is effectively dead, and only a few elderly men still wield the hand tattoo used to mark a successful headhunter (Freeman, 1970; Laukien, 2005). Iban engage in far-flung wage labor opportunities alongside members of other ethnic groups with which they have prior histories of war (Lumenta, 2003). They seek formal education, consume Malaysian mass media, and many have converted to dominant world religions, including Christianity and Islam. Many Iban now also identify as citizens of Malaysia in addition to being Iban (Lumenta, 2003; Postill, 2006). At times, violence reminiscent of earlier times flares up (BBC News, 2001), but after two centuries, most Iban have a very different way of defining insiders and outsiders and very different views about appropriate social behavior with other groups.

The Iban transformation illustrates three points. First, the ways that people behave toward others can depend heavily on how those others are classified—as kin, friends, and community members or outsiders, strangers and foreigners. Second, human populations can vary dramatically in: (1) how they define closeness and distance of a social partner and (2) how these qualities of a partner influence social behavior. Third, these population differences are not fixed or static. Populations can change quite dramatically within several generations, in this case, from hunting the heads of neighboring groups to participating relatively peacefully in a much larger nation-state and world system.

How people socially and psychologically construct boundaries between insiders and outsiders or plot gradients of social distance and how these models of boundaries and distance shape behavior toward others are critical questions for a number of fields. Current models for the evolution of human social behavior, and of large-scale cooperation specifically, rely on the construction of groups that can contain the fruits of cooperation, exclude outsiders, and compete with other groups (Boyd et al., 2003; Choi and Bowles, 2007). Paradoxically, the same tribal instincts that may have fostered the human capacity for large-scale cooperation today pose problems for building peaceful and just societies at ever larger scales (Bernhard et al., 2006; Richerson and Henrich, 2012). They also underlay many currently recognized problems in today's world, including favoritism, racial and ethnic discrimination, armed ethnic conflict, and genocide (Levine and Campbell, 1972).

In the past decade, researchers have proposed a number of theories to account for these population differences in parochialism and to explain historical changes like those observed among Iban. However, these diverse approaches are relatively scattered across the social and behavioral sciences, they encompass a wide range of motivations and behaviors under the broad rubrics of in-group favoritism, ethnocentrism, xenophobia, and parochial altruism, and these different theories rarely come into contact in the same paper or analysis. In this paper, we clarify the diverse ways that scholars have operationalized parochialism, we outline and synthesize current hypotheses for cross-population variation in parochialism, and we discuss key methodological challenges in assessing these diverse economic and evolutionary hypotheses.

## Varieties of parochialism

Humans do not have a general tendency to help, protect, or harm others. Rather, these behaviors are conditioned by many contextual factors (Bekkers and Wiepking, 2011), including the perceived need of the recipient (Taormina and Messick, 1983; Engel, 2011), the legitimacy of the request for help (Bickman and Kamzan, 1973), the degree to which someone deserves harm or help (Skitka and Tetlock, 1992), genetic relatedness or kinship with a person (Rachlin and Jones, 2008; Alvard, 2009), and whether the individual or group are perceived to pose a threat (Semyonov et al., 2004). The degree to which an actor feels socially close to another individual also reliably guides social behavior, whether social closeness is determined by subjective assessments of a spatial metaphor (e.g., closeness or insideness) or by common membership in a group (Leider et al., 2009; Goeree et al., 2010; Mathew and Boyd, 2011; Branas-Garza et al., 2012). Here, we refer to the broad tendency to rely on cues of social closeness in guiding behavior as *parochialism*, a concept which encompasses a number of related concepts including xenophobia, ethnocentrism, and parochial altruism.

The social and behavioral sciences have a long tradition of studying the proximate mechanisms by which social closeness and group membership influence behavior toward others and how groups emerge in experimental settings (Sherif, 1961; Tajfel et al., 1971; Brewer, 1979; Glaeser et al., 2000; Hewstone et al., 2002; Dovidio et al., 2005; Goette et al., 2006). All of these approaches are united in studying how our decisions to help, protect or harm someone are shaped by perceptions of social closeness. However, these approaches also differ in two key respects: (1) in how social closeness is operationalized, and (2) in what behaviors, preferences and motivations are considered. We review these differences here.

### Operationalizing social closeness

Social closeness has been operationalized as both an ordinal and categorical concept. As an ordinal concept, researchers have assessed social closeness to a partner or a group in several ways, by asking participants: (1) to rate partners on a Likert scale in terms of “emotional closeness,” “we-ness,” or spatial overlap (Aron et al., 1992; Myers and Hodges, 2012), (2) to rank partners in terms of relative closeness (Rachlin and Jones, 2008), and (3) to indicate to what degree one sees oneself as a member of a group (Inglehart et al., 2006). A spatial metaphor is used to describe and assess this concept in many, but not necessarily all languages (as in English, Hruschka, 2010).

Operationalized as a categorical concept, social closeness is based on participation in a relationship (e.g., close friend, family) or on membership in a common group. This can be operationalized categorically in terms of the existence of a recognized face-to-face relationship, including different kinds of kinship, friendship, and acquaintanceship (Hruschka, 2010). It can also be operationalized categorically in terms of common membership in a larger group, such as a religion, denomination, nationality, region, city, neighborhood, language, university, ethnicity, or race (Hruschka and Henrich, 2013).

### Behaviors, preferences and motivations

Parochialism is manifest in a number of behaviors, preferences and motivations, which we classify here as avoidance, trust, favoritism, permission to harm, and ingroup bias.

First, one can accept or avoid individuals of different groups in everyday interaction (henceforth, *avoidance*). One of the first attempts to assess parochialism, the Bogardus social distance scale, used this approach by asking how much a respondent would accept someone from another ethnic or religious group as a close relative by marriage, as a close personal friend, as a neighbor on the same street, as a co-worker, as a fellow citizen, and as a visitor to one's country (Bogardus, 1933; Inglehart et al., 2006). Second, social closeness correlates with how much people report trusting others. This creates different “radii of trust,” where people generally report trusting family members more than personally known others and neighbors, who in turn are trusted more than individuals from other regions, ethnicities and countries (Allik and Realo, 2004; Whitt, 2010; Delhey et al., 2011). Third, social closeness can influence how we distribute resources or protect others (*favoritism)*, whether in allocating jobs (Van de Vliert, 2011) or money (Fershtman and Gneezy, 2001; Bahry et al., 2005; Habyarimana et al., 2007; Whitt, 2010), violating a rule to help others (Trompenaars and Hampden-Turner, 2000; Hruschka et al., submitted) or acting to protect others (Bernhard et al., 2006). Fourth, social closeness can shape how morally acceptable it is to harm others or how hostile one feels toward others (*permission to harm*) (Sutlive, 1992; Cashdan, 2001; Mathew and Boyd, 2011). Fifth, people tend to rank socially close friends, family and community members as better than others. This *ingroup bias* can be expressed as pride in family or country or relative ratings of competence, intelligence, or other positive qualities (Brown, 1986; Evans and Kelley, 2002). Researchers have measured these different behaviors, motivations and preferences in several ways, as self-reported attitudes (Evans and Kelley, 2002), behavior in hypothetical scenarios (Trompenaars and Hampden-Turner, 2000; Whitt, 2010), behavior with real monetary stakes (Fershtman and Gneezy, 2001; Bahry et al., 2005), and real-world behavior (Gazal-Ayal and Sulitzeanu-Kenan, 2010).

In addition to these specific manifestations of parochialism, researchers have also deployed several general measures derived from factor analyses intended to capture investment in one's local group. Perhaps the best known measure is collectivism, or the tendency to care about the consequences of one's behavior for in-group members and to be willing to sacrifice personal interests for collective gains (Triandis et al., 1988; Hofstede, 2001). Schwartz's measure of embeddedness also falls into this category and captures restraint of actions or inclinations that might disrupt group solidarity or the traditional order (Schwartz, 2006).

Little research has focused on how these diverse measures of parochialism covary across individuals and populations. In a sample of 186 small-scale societies, between-society variation in hostile attitudes toward other ethnic groups was not correlated with the degree of belonging to one's own ethnic group (Cashdan, 2001). However, a number of measures of avoidance, favoritism, and ingroup bias are highly correlated across countries, and these also correlate with other non-specific measures of collectivism and embeddedness (Hruschka and Henrich, 2013). Interestingly, the tendency to favor socially close others appears to extend across diverse social scales, all the way from family to nation. For example, increased population levels of parochialism at one level (e.g., the immediate family) are moderately to strongly associated with parochialism at other levels (e.g., extended relatives, friends, compatriots) (Hruschka and Henrich, 2013). Measures of parochialism also appear to be associated with a more general syndrome of social and psychological tendencies, including tighter adherence to norms (Gelfand, 2011), greater concerns about obedience and authority (Inglehart et al., 2006), greater religiosity (Fincher and Thornhill, 2012), and more concerns about purity violations (Haidt and Graham, 2007).

Thus, many measures of in-group favoritism appear to correlate, although out-group hostility may constitute an independent dimension (Cashdan, 2001). Parochialism at one social scale (e.g., immediate family) appears to be associated with parochialism at other scales (e.g., extended family, community, and country). And parochialism appears to be one part of a syndrome of other tendencies toward conformity and obedience.

## Cross-population variation in parochialism

In the last two decades, psychologists and economists have begun to identify key cognitive and neurobiological mechanisms underlying parochialism, including perceptions of threat (Reik et al., 2006) and the role of oxytocin and brain circuits in modulating behavior toward in- and out-group members (De Dreu et al., 2010; Baumgartner et al., 2011; De Dreu, 2012). Researchers have also identified specific kinds of activities which can increase social closeness to others, including focused conversations (Aron et al., 1997), synchronized movement (Vacharkulksemsuk and Fredrickson, 2012), and synchronized multisensory inputs (Paladino et al., 2010). Moreover, it appears that the capacity and propensity to differentiate social groups arises early in development (Kinzler et al., 2007). However, researchers have only recently begun to explore why these psychological capacities for parochialism are recruited differently in different human populations and across different cultural settings (Miller and Bersoff, 1998; Buchan et al., 2009; Gelfand, 2011; Van de Vliert, 2011; Fincher and Thornhill, 2012; Hruschka and Henrich, 2013; Hackman and Hruschka, 2013b).

There are several ways that populations differ in parochialism. First, what counts as a kin tie, a friendship, or an in-group and what counts as appropriate behaviors with different social partners is informed by local cultural categories and norms. For example, most populations in the US do not have a cultural category of blood brother, and so there is no clear set of norms or expectations applied to being in such a relationship (Hruschka, 2010). Second, the social techniques available to organize and maintain in-groups of varying sizes and scales constrain the kinds of in-groups to which people can belong. Mass media and formal schooling makes it much more likely that people can identify with groups as large as those encompassed by modern nation-states. World religions disseminate and enforce common languages, symbols, and rituals which can forge large populations into a single in-group (Atran and Henrich, 2010). These social techniques permit the creation of new in-groups that may have never been possible before. Third, the most salient in-group category can change quickly based on local practices and contexts. Among Enga horticulturalists in Papua New Guinea, rituals aimed at dehumanizing members of another group can swiftly recast allies as enemies (Wiessner, 2006), and among the Nuer of Sudan, changing patterns of competition over resources can re-align in-groups and out-groups (Evans-Pritchard, 1940). Finally, and most relevant to this article, given in-groups of similar scales, individuals from different populations differ remarkably in several crucial ways, including how much they trust and avoid outsiders and how much they favor friends, family, and community members (Fukuyama, 1995; Inglehart et al., 2006; Delhey et al., 2011; Hruschka and Henrich, 2013).

From the perspective of neurobiology, cross-population variability provides an opportunity to establish and distinguish those aspects of human brains and psychology that are reliably developing products of pan-human genes from those that depend on particular culturally-constructed niches (e.g., institutions) or ecological conditions. Grounded in culture-gene coevolutionary theory (McElreath et al., 2003; Henrich and Henrich, 2007), there is now substantial cross-population and developmental evidence suggesting that humans come equipped with cognitive abilities and psychological motivations to preferentially attend to, learn from, and interact with co-ethnics—individuals who share their markers for dress, dialect, language and bodily ornamentation. For example, infants and young children from diverse societies readily use dialect and dress to distinguish in-group members/coethnics (Kinzler et al., 2011, 2012; Mahajan and Wynn, 2012; Corriveau et al., 2013). On the basis of these rather sparse cues, infant and children preferentially learn from these individual (Shutts et al., 2013), seek interaction with them, and punish them for norm-violations (Schmidt et al., 2012). As expected from the theory, such ethnic cues can even trump racial differences in both young children (Kinzler et al., 2009; Corriveau et al., 2013) and sometimes in adults (Kurzban et al., 2001).

However, such reliably developing features of human cognition and motivation have to be understood in the light of two emerging lines of theory and evidence. First, growing up and ontogenetically adapting to very different environments means that different populations of humans have different brains and biologies, even when no genetic differences exist between populations (Henrich et al., 2010b,c). Within neuroscience, both training studies and cross-population research indicates that brains and bodies develop somewhat differently, in different environments, and yield distinct patterns of activation and hormonal responses to identical stimuli (Nisbett and Cohen, 1996; Kitayama et al., 2009; Woollett and Maguire, 2011). Second, mounting evidence indicates that cultural evolutionary processes have systematically shaped the physical and social (institutional) environments that developing humans face. This implies that cultural evolution has shaped our brains ontogenetically and over historical time (Henrich et al., 2012; Richerson and Henrich, 2012). For example, behavioral studies of children from ages 3 to 14 and adults across six diverse societies, ranging from Congo foragers to Westwood Los Angelenos, reveals the emergence of distinct developmental trajectories for social behavior in different places (House et al., 2013). This pattern is broadly consistent with the presence of market institutions in these societies. Several theories suggest that cultural evolution has harnessed and extended aspects of our innate parochialism in forming nations and religions.

These developments suggest that, rather than attempting to make potentially dubious inferences by generalizing from WEIRD undergraduates (Chiao and Cheon, 2010; Henrich et al., 2010c), neuroscientists need to develop collaborations that take advantage of both the existing theories discussed in this paper and then tap the now well-establish psychological diversity in our species.

## Theories of cross-population variation in parochialism

Several theories have been proposed to account for cross-population differences and historical changes in parochialism. These theories vary along two major axes. First, they vary in the specific mechanisms by which individuals and populations change in response to their environment. Second, they vary in the specific ecological and social conditions which are posited to shape parochialism. We first review proposed mechanisms and then outline the different proposals for relevant environmental conditions, including market integration, religion, and environmental uncertainty.

### Mechanisms

Parochial behaviors and motivations might change in response to the environment in several ways. These include genetic adaptation, learning over development, immediate facultative responses, and social learning (Schaller and Murray, 2010).

One recent example of a genetic mechanism is Chiao and Blizinsky's proposal that differences in collectivism may result from allelic variation in the serotonin transporter functional polymorphism (5-HTTPLOR). Specifically, collectivist nations had higher frequencies of the short allele which is associated with heightened anxiety, harm avoidance, fear conditioning, and attentional bias to negative information (Chiao and Blizinksy, 2010). Furthermore, their analyses suggested that these genetic differences may reflect adaptations to infectious disease prevalence. However, a re-analysis of these data suggests that their findings can be accounted for by a model of neutral genetic and cultural change with migration (Eisenberg and Hayes, 2011).

At short time scales, individuals may respond relatively immediately to changing environmental conditions. For example, a vast body of experimental work indicates that cuing uncertainty in a number of domains, including mortality, disease, and social exchange, makes people more likely to favor in-group members (Kollack, 1994; Navarrete et al., 2004; Heine et al., 2006; Hohman, 2011). Conversely, priming individuals with terms related to safety and security make them less likely to favor in-group members (Mikulincer and Shaver, 2001). Thus, parochial motivations and behaviors can respond quite rapidly to environmental cues.

At longer time scales that are still shorter than a lifespan, parochial motivations and behaviors may change in response to environmental cues during specific windows of development. For example, Fincher and Thornhill propose that individual's may learn about disease risk from the local environment through recurring immune system activation, which in turn affects social behaviors and motivations (Fincher and Thornhill, 2012). Recent studies of exposure to war, suggest that specific parochial motivations and behaviors are sensitive to violence between ages of 7 and 20, but not before or after that window (Bauer et al., forthcoming). In addition to direct learning through exposure to their environment, individuals may also learn from others about key aspects of the environment, such as local disease risk, threat of mortality, and risk of inter-group conflict (Fincher and Thornhill, 2012).

In addition to learning environmental cues which may shape parochialism, individuals may also learn relevant social norms about who are members of one's in-group and how one should treat insiders and outsiders under different conditions (Henrich et al., 2010a). For example, individuals frequently engaging in market interactions may learn and eventually internalize norms about dealing fairly with relative strangers and anonymous others (Henrich et al., 2010a).

Each of these mechanisms would lead to different expectations about the time scale of response, from months, to decades, to centuries (Schaller and Murray, 2010). Apparent behavioral fit with specific environments may also result from a combination of co-evolutionary feedback loops involving these mechanisms. For example, infectious disease risk, which is proposed by some theories to be a driver of parochialism, is not simply an exogenous element of the environment. Rather it has changed in response to the emergence of public health institutions, which were in turn the outcome of early large-scale collective attempts to improve others' health. Such feedback between environments and behavior can lead to significant co-evolutionary trajectories.

### Market integration

The market integration hypothesis proposes that market norms emphasizing fair treatment of anonymous others have culturally evolved to sustain mutually beneficial exchanges in contexts demanding frequent interaction with strangers or ephemeral interactants. As, individuals increasingly interact with markets, they adopt and internalize these norms, and markets spread more successfully in places where such norms are already in place (Henrich et al., 2010a). Thus, individuals with greater market exposure will be more likely to have adopted or internalized these norms and thus will treat anonymous others more fairly. This hypothesis has been tested, replicated, and extended in two separate projects covering 24 different societies from Siberia to New Guinea. Overall, more market integrated societies tend to split pots of money more evenly with anonymous others, independent of the threat of punishment, income, wealth, education, community size, sex, and age (Henrich et al., 2005, 2010a). Since such equitable behavior arises even when punishment is not possible, and anonymity is assured, the authors argue it is guided by internalized local norms. More recent studies among 57 communities in Ethiopia which are tied to their land by customary rights suggests that the relationship between market integration and prosocial behavior with anonymous others is not due to selective migration (Rustagi et al., 2010; also see Voors et al., 2012 for findings from Burundi). And, recent experimental work on “giving” by Westerners show that such responses are automatic (Rand et al., 2012) and rely on the brain's reward circuitry (Fehr and Camerer, 2007; Harbaugh et al., 2007), suggesting that they do reflect internalized patterns of behavior.

### Religion

Many religious traditions emphasize the importance of helping strangers and treating others fairly, and thus enculturation in specific religions may reduce parochialism—either within one's religion or even across religions. One current theory holds that modern world religions, such as Christianity and Islam, were able to spread precisely because they effectively enculturated norms of prosocial behavior which galvanized large-scale cooperation among relatively anonymous strangers (Atran and Henrich, 2010). According to this view, followers of modern world religions, such as Christianity and Islam, will be more likely to have internalized these norms of prosocial behavior and will thus treat anonymous others with greater fairness and generosity. Findings from the cross-society studies described earlier are also consistent with this hypothesis (Henrich et al., 2010a), showing that adherents to modern world religions offer more in bargaining experiments. Similar experiments among Western populations have shown that unconsciously priming Christians, but not atheists, with “God” causing them to be more equitable in bargaining games, cheat less, cooperate more and sometimes punish selfishness to a greater extent (Randolph-Seng and Nielsen, 2007; Shariff and Norenzayan, 2007; Ahmed, 2009; McKay et al., 2011; Laurin et al., 2012).

World religions may also exhibit variation in how strongly they affect parochialism. Experiments meant to measure trust in anonymous transactions show that religious people are trusted more, especially by other religious people. Consistent with this, work from psychology suggests Christians trust each other more because they believe other Christians know God is watching (Gervais et al., 2011). Ritual participation seems to have effects independent of belief in God: participation in rituals increases in-group favoritism, in the form of cooperation (Sosis and Ruffle, 2003; Ruffle and Sosis, 2006), and is associated with support for out-group aggression (Ginges et al., 2009).

Protestantism may be of particular interest here. Weber, and more recently Fukuyama, have argued that a key effect of Protestantism was to “shatter the fetters” of the extended family (Weber, 1951; Fukuyama, 2011), and recent authors have pinned this on Protestant core values of self-reliance and individualism which potentially led to less investment in family, friends, and local in-groups (Lipset and Lenz, 2000; Treisman, 2000). Consistent with this, cross-national analyses show that majority Protestant countries consistently report less favoritism, in-group bias, and out-group avoidance, after adjusting for economic security and government effectiveness, than countries with other religions in the majority—including Orthodox Christianity, Catholicism, and Islam (Hruschka and Henrich, 2013).

### Globalization

The globalization hypothesis proposes that as people are increasingly exposed to individuals outside their community through new forms of mass media, including newspapers, the internet, social media, television, and movies, and through new forms of social interaction, they are less likely to think in terms of in-groups and out-groups and more likely to imagine humankind as a “we” where there are no “outsiders” (Buchan et al., 2009). Thus, individuals with greater interactions with global communication (e.g., televisions, print media, and employment in transnational firms) will be more inclined to engage in collective action with individuals outside of their immediate in-group. This hypothesis overlaps with the market integration hypothesis, but proposes that many kinds of interactions, including mere exposure to people from other countries through mass media, can change responses to outsiders. Consistent with this hypothesis, Buchan et al. (2009) found that contribution to global public goods increases with increasing exposure to different forms of mass media.

### Existential or material security hypotheses

Here we group three related hypotheses that focus on the effects of various form of material or existential security on individual decision making, development, and cultural evolution. The first, generalized insecurity, casts a broad net by proposing that insecurity will influence parochialism, while the others suggest that individuals respond selectively to specific kinds of threats, such as pathogens, inter-group conflict, and thermic stress.

#### Generalized insecurity

Variants of the institutional quality hypothesis propose that informal and formal institutions change the costs and benefits of parochialism, which in turn shape social norms and behavior by a number of potential mechanisms. Public services, global markets, and social safety nets that mitigate material threats and guarantee safe interaction with anonymous partners may render investments in an expansive network of kith and kin less necessary as alternative forms of social insurance. It may also foster greater interaction and trust with a larger set of individuals (Inglehart and Welzel, 2005; Inglehart et al., 2006; Hruschka, 2010; Hruschka and Henrich, 2013). Ample experimental and observational evidence demonstrates the role of economic, existential, and symbolic security on parochial attitudes and behaviors (Kollack, 1994; Navarrete et al., 2004; Heine et al., 2006; Canetti-Nisim et al., 2008; Proulx and Heine, 2010; Hohman, 2011; Kaplan et al., 2012). Conversely, priming individuals with terms related to safety and security make them less likely to favor in-group members (Mikulincer and Shaver, 2001). And a body of work in political science and economics has examined how norms and institutions reduce barriers to trust, encourage cross-group cooperation and discourage parochialism in ethnically-divided societies (Knight, 1992; Jackman and Miller, 2004; Whitt, 2010). Several lines of observational evidence are also consistent with this hypothesis that stronger institutions and less exposure to generalized risk of famine, disease, and inter-group conflict are associated with reduced in-group favoritism (Cashdan, 2001; Inglehart et al., 2006; Whitt, 2010; Hruschka and Henrich, 2013).

#### Pathogen stress

The above hypothesis proposed that parochialism responds to existential or material insecurity, in general. However, there are other, more domain-specific, hypotheses that propose that specific forms of insecurity may have parochial effects. Recently, several evolutionary researchers have proposed that parochialism constitutes a form of behavioral immune system against the spread of pathogens. According to this hypothesis, in regions with high risk of infection by dangerous pathogens, individuals will preferentially interact with in-group members in a way that insulates them from infection by out-group members (Schaller and Murray, 2010; Fincher and Thornhill, 2012). Though originally predicting avoidance of and hostile attitudes toward out-groups, the theory has been extended to account for other aspects of parochialism as well, including ingroup favoritism and bias (Fincher and Thornhill, 2012). This hypothesis differs crucially from other hypotheses by positing that the adaptive mechanisms responsible for this effect are specific to pathogen risk and were designed to impede the spread of pathogens or to provide social support specifically in case of infection. Different mechanisms have been proposed, including sensitivity to immune system activation, social learning of local disease risks and direct observation of parasitic infections, all of which would lead to relatively fast facultative responses. Other longer-term mechanisms include culturally evolutionary processes by which groups which have social norms preventing and mitigating threats of infection (e.g., parochial social interaction) are more likely to spread and persist in regions of high endemic pathogen threat (Schaller and Murray, 2010).

Emerging experimental evidence suggests that people do indeed adjust some social motivations and behaviors (i.e., conformism) to specific cues of pathogen threats over and above generalized threats (Murray and Schaller, 2012). However, cross-national and cross-state studies have shown mixed support for this hypothesis as an explanation for extant cross-population variation in parochialism (Currie and Mace, 2012; Fincher and Thornhill, 2012; Cashdan and Steele, 2013; Hruschka and Henrich, 2013; Hackman and Hruschka, 2013a; Hruschka et al., submitted).

#### Inter-group conflict hypothesis

Another insecurity hypothesis focuses narrowly on how the threat of, or experience of, intergroup conflict may strengthen in-group preferences, including egalitarianism. Using simple choice tasks in two post-conflict societies, the Republic of Georgia and Sierra Leone, Bauer et al. (forthcoming) show that the experience of war creates an enduring increase in individuals' in-group egalitarian motivations, while not influencing their motivations toward out-group individuals. However, the effect of war only left an enduring mark on motivation if individual experienced the war during a developmental window from roughly age 7 to 20. The effect of war experience had no impact on those under about age 7, and only small effects on those who experience the war past roughly age 20. These results are supported by other work showing that senior Israeli citizens were more willing to punish norm-violators in a bargaining game during the conflict with Hezbollah, compared to both pre- and post-war measures (Gneezy and Fessler, 2012). Working in Burundi, Voors et al. show that victimization in war increases people altruism toward their neighbors, as well as their temporal discounting and risk preferences. This work also examines the effects of non-war related shocks to security, including draught, flooding, and pestilence. This work shows that the experience of droughts also increased altruism toward in-group members, an independent effect, but did not alter temporal discounting or risk preferences. This suggests that war-related insecurity vs. drought-related insecurity may produce somewhat different psychological effects (Voors et al., 2012), supporting the notion that these are distinct domains. However, aside from this finding, all of these data are also consistent with the generalized insecurity hypothesis.

#### Thermic stress hypothesis

The climate-economics hypothesis proposes that much of human culture is an adaptive response to thermic stress—either extreme cold or extreme heat—but that this can be buffered by economic resources. In the case of in-group favoritism, Van der Vliert argues that populations facing extreme temperature stress without the economic resources needed to adapt to that stress respond psychologically in a number of ways, including greater preferences for authoritarian leadership and for favoring members of one's in-group (Van de Vliert, 2011; Van de Vliert and Postmes, 2012).

## Methodological issues in assessing cross-population hypotheses

In the last decade, the observation of substantial between-population differences in parochialism has inspired considerable theoretical work on the possible causes of these between-population differences. This is exciting progress, and this review describes a number of promising theories that may account for cross-population variation.

However, there are serious challenges in efforts to discriminate between these different hypotheses and to identify the specific mechanisms by which parochialism rises and falls in societies. Most studies have relied on observational cross-population designs, raising concerns about causality, identification of specific mechanisms, the direction of effects, and the time-scale of adaptation. Several strategies can provide some check against these issues.

The first task is to begin culling hypotheses through strategic model comparison rather than testing each hypothesis against a straw man null model. This involves identifying different predictions across models and then finding appropriate cross-population data which can discriminate between these predictions. For example, in a recent study of population-level parochialism across countries, Hruschka and Henrich directly compared the parasite stress hypothesis with the material insecurity hypothesis using novel checks against regional autocorrelation, new longitudinal data to assess reverse causation, and an instrumental variable to check for the effects of omitted variables. These results provided consistent support for the material insecurity hypothesis. It also challenged prior studies supporting the parasite stress hypothesis which had not included these methodological checks. In another paper, Hackman and Hruschka re-assessed analyses of US data which had previously found an association between pathogen stress and collectivism. With new data stratified by race, they showed the observed associations across states were due exclusively to substantial differences across US Whites and US Blacks. They also found support for an alternative hypothesis related to the material insecurity hypothesis (Hruschka and Henrich, 2013; Hackman and Hruschka, 2013a). Of course, such model comparison using observational data does not definitively show that the “winning” hypothesis is correct. However, it helps winnow the playing field.

Another important check can come from combining psychological experiments with cross-population studies in order to triangulate between potential psychological processes and the macro-scale correlates of cross-population variation. The findings of experiments alone may not scale up easily to account for cross-population differences, and cross-population correlations without grounding in established psychological mechanisms can easily be explained away as spurious associations. Integrating these two orders of data can ensure that hypotheses are consistent at both the individual and population level. A number of theories, including the market integration, religion, institutional quality, and pathogen stress hypotheses have begun to accrue data at both of these levels.

To mitigate some concerns about causality, mechanism, and directionality, the social sciences offer a number of tools that provide further checks on findings from cross-population observational data. Instrumental variable analysis commonly used in economics provides one additional check by identifying quasi-experimental assignments in observational data. For example, Acemoglu et al. used the mortality rates of early settlers in European colonies (1600–1875) as an instrumental variable which is expected to affect contemporary government effectiveness—an important variable in the material security hypotheses of parochialism. There is ample historical evidence that European colonizers avoided settling in places with high mortality rates, such as in the Belgian Congo (McNeill, 1977; Acemoglu et al., 2001), and instead of settling, they set up extractive systems. In situations of low mortality, on the other hand, colonizers settled in larger numbers and brought with them institutions, such as respect of private property, checks and balances in government, and equality of opportunity, which in turn fostered greater government effectiveness that persisted even after independence (Acemoglu et al., 2001). These measures of settler mortality act in some ways as quasi-experimental assignments of countries to different levels of government effectiveness, and Acemoglu et al. used this quasi-experimental assignment to examine the effect of government institutions on economic growth. More recently, Hruschka and Henrich have used the same reasoning to examine the effect of government institutions on parochialism (Hruschka and Henrich, 2013).

As access to longitudinal data increases with longer running cross-national surveys, it will also be possible to assess the temporal precedence and coincidence of different changes within populations (Inglehart et al., 2006; Hruschka and Henrich, 2013). For example, between 1925 and 2005, US samples have shown steadily decreasing avoidance of other ethnic groups in a number of domains—as in-laws, friends, neighbors, and fellow citizens (Bogardus, 1933; Parrillo and Donoghue, 2005). Long-term longitudinal data like this may provide insights into what factors most readily account for long-term changes in parochialism and how rapidly changes occur. Migration studies, originally developed in epidemiology, but now applied in economics, also show some promise in identifying the time-scale by which different aspects of parochialism change across generations who are put into novel contexts (Guiso et al., 2006; Fisman and Miguel, 2007; Giuliano and Alesina, 2010). For example, Giuliano and Alesina used such a design to show that second generation immigrants carry “cultural baggage” from their home country. Specifically, even after two generations, immigrants from countries with greater stated investment in family ties moved less and lived with their parents longer (Giuliano and Alesina, 2010).

Another approach is to look for natural experiments, as Bauer et al. did with their investigation of the effects of war on parochialism (Bauer et al., forthcoming). They looked around the globe for situations in which the effects of war on individuals, households, and communities were—at least plausibly—random with respect to individuals' own parochial motivations. Refugees and soldiers would be relatively easier to access compared to the approach they took, but both fleeing and being alive might be caused by their particular social motivations (therefore endogenous). As checks on the natural experiment assumption, they also (1) examined whether observables, like ethnicity or age, predicted experiencing war (they did not) and (2) performed their analyses just on those who were children at the time of the conflict (and thus have less control). These analyses support the idea that the experience of war was imposed exogenously, and thus provides a natural experiment.

Despite all of these possible checks and triangulations, observational data is still plagued by concerns about endogeneity and non-random assignment of cases which can threaten causal interpretations. Thus, once hypothesis are culled and honed through the above-mentioned techniques, a growing body of field experiments in economics, public health, and development currently used to understand health and development holds promise in assessing specific mechanisms by which economic, social, and environmental conditions inhibit or foster parochialism (Banerjee and Duflo, 2011). With this combination of model comparison, cross-level confirmation, statistical checks on temporal precedence and causality, and ultimately field experiments of different hypotheses, this exciting and crowded field of theories for parochialism will hopefully lead to a clearer understanding of the specific mechanisms and time scales by which population differences in parochialism emerge and sustain themselves.

### Conflict of interest statement

The authors declare that the research was conducted in the absence of any commercial or financial relationships that could be construed as a potential conflict of interest.
